# Complete Genome Sequence of a Novel Mutation of Seoul Virus Isolated from Suncus murinus in the Fujian Province of China

**DOI:** 10.1128/genomeA.00075-15

**Published:** 2015-04-02

**Authors:** Yu-ping Wang, Xiao-long Zhang, Jian-min Zhang, Xiao-mei Cao, Hui Han, Ye-neng Huang, Hai-ling Wang, Min Chen, Bo Gao, Li-si Yao

**Affiliations:** a Institute of Health, Chinese Academy of Inspection and Quarantine, Beijing, China; b Fujian Entry-Exit Inspection and Quarantine Bureau, Fuzhou, China

## Abstract

Suncus murinus has been identified as the host for Seoul virus (SEOV). Here, we report the complete genome sequence of SEOV strain Fj372/2013, which was isolated from the lung tissue of Suncus murinus in the Fujian Province of China. A mutation A38C was observed in an open reading fragment of the middle segment.

## GENOME ANNOUNCEMENT

Hemorrhagic fever with renal syndrome (HFRS) is caused by hantavirus. During 2006 to 2012, a total of 77,558 HFRS human cases and 866 deaths were reported in China (1). Seoul virus (SEOV) is one of the main etiological agents for HFRS, which causes a moderate form of HFRS (2). As with other members of the family Bunyaviridae, hantaviruses are enveloped, negative-sense RNA viruses. The genome consists of three segments, designated large (L), medium (M), and small (S). They encode RNA-dependent RNA polymerase, the glycoprotein precursor protein that is processed into 2 separate envelope glycoproteins (Gn and Gc), and the nucleocapsid (N) protein, respectively (3, 4).

As Rattus species are cosmopolitan, SEOV has the potential to cause human disease worldwide (5). SEOV is mainly associated with the brown rat (Rattus norvegicus) and the black rat (Rattus rattus) throughout the world. Suncus murinus (Asian house shrew) is an insectivore. Two reports were confirmed that SEOV infected Suncus murinus in China (6, 7). However, no complete genome sequence of SEOV isolated from Suncus murinus has been previously reported*.*

Here we report the complete genome of strain Fj372/2013. The SEOV was isolated from the lung tissue of Vero-E6 cells of Suncus murinus in the Fujian Province of China. Standard reverse transcription-PCR was performed with primers specific for SEOV. The genome sequence was determined by using overlapped consensus primers and direct sequencing (8, 9). All sequencing was carried out bidirectionally. The full-length genome sequence was established by assembling overlapping fragments with the SeqMan program of the Lasergene 8.1 package (DNAStar).

The genome of SEOV strain Fj372/2013 comprised three negative-stranded RNA segments referred to as S, M, and L. The full lengths of the segments are 1,769, 3,605, and 6,530 nucleotides, respectively. The three genes encode proteins with the following amino acid lengths: nucleocapsid, 429; glycoprotein precursor, 1,134; and transcriptase protein, 2,152, respectively. Sequence analysis of L, M, and S segments showed that Fj372/2013 has 95.7%, 95.7%, and 96.5% nucleotide identities to SEOV strain 80-39 isolated in South Korea, and 97.5%, 97.5%, and 96.1% to strain ZT10 in China, respectively. A mutation (A38C) was observed in an open reading fragment of the M segment, and the deduced amino acid is changed from Gln to Pro in position 13 of Gn. The highly conserved sequence motif WAASA (10) is located in amino acids 642 to 646 of Gn. Phylogenetic analysis based on M and S segments, which was performed by the neighbor-joining method and maximum likelihood method using MEGA5.22 (11), showed that Fj372/2013 belongs to the clade A subtype (4).

The sequence information provided here is useful for understanding the circulation and evolution of SEOV in China.

### Nucleotide sequence accession numbers.

The whole-genome sequence of SEOV strain Fj372/2013 isolated in Fujian Province in 2013 has been deposited in GenBank under the accession numbers KP645196 for the L segment, KP645197 for the M segment, and KP645198 for the S segment.
